# Non-Coding RNAs: The “Dark Matter” of Cardiovascular Pathophysiology

**DOI:** 10.3390/ijms141019987

**Published:** 2013-10-09

**Authors:** Claudio Iaconetti, Clarice Gareri, Alberto Polimeni, Ciro Indolfi

**Affiliations:** Division of Cardiology, Magna Graecia University, URT Consiglio Nazionale delle Ricerche (CNR), Catanzaro 88100, Italy; E-Mails: iaconetticlaudio@unicz.it (C.I.); c.gareri@unicz.it (C.G.); polimeni@unicz.it (A.P.)

**Keywords:** non-coding RNA, microRNA, long non-coding RNA, vascular development, vascular disease, heart pathophysiology

## Abstract

Large-scale analyses of mammalian transcriptomes have identified a significant number of different RNA molecules that are not translated into protein. In fact, the use of new sequencing technologies has identified that most of the genome is transcribed, producing a heterogeneous population of RNAs which do not encode for proteins (ncRNAs). Emerging data suggest that these transcripts influence the development of cardiovascular disease. The best characterized non-coding RNA family is represented by short highly conserved RNA molecules, termed microRNAs (miRNAs), which mediate a process of mRNA silencing through transcript degradation or translational repression. These microRNAs (miRNAs) are expressed in cardiovascular tissues and play key roles in many cardiovascular pathologies, such as coronary artery disease (CAD) and heart failure (HF). Potential links between other ncRNAs, like long non-coding RNA, and cardiovascular disease are intriguing but the functions of these transcripts are largely unknown. Thus, the functional characterization of ncRNAs is essential to improve the overall understanding of cellular processes involved in cardiovascular diseases in order to define new therapeutic strategies. This review outlines the current knowledge of the different ncRNA classes and summarizes their role in cardiovascular development and disease.

## 1. Introduction

Many studies have recently focused on understanding RNA metabolism and its implication in development and disease processes. Genomic tiling arrays and RNA-Sequencing have showed that the human genome is dynamically transcribed and leads to the production of a complex world of RNA molecules of which only a small fraction is translated into proteins [1]. In fact, application of high-throughput sequencing technologies in the analysis of mammalian transcriptomes, revealed a wide spectrum of RNA molecules that do not encode protein, termed non-coding RNAs (ncRNAs) [2]. For many years the role of these molecules remained unknown, so ncRNAs were called the “Dark Matter” of biology. To date many studies have been carried out on these molecules, especially on microRNAs, partially clarifying their roles. However many mechanisms and functions of different classes of ncRNA still remain unknown. Emerging evidence indicates that the non-coding portion of the genome is critical in the regulation of multiple biological processes, such as differentiation, development, post-transcriptional regulation of gene expression and epigenetic regulation [3–5]. Recently, many classes of ncRNA have been described to be associated with human disease [6]. Cardiovascular disease is a major cause of mortality and hospitalization worldwide [7], and the work of multiple research groups has been devoted to determine the molecular mechanism underlying heart and vascular disease. Recent studies indicate that altered ncRNA expression and function have been strongly implicated in cardiovascular disease such as myocardial infarction, cardiac hypertrophy and coronary artery disease [8–10]. The transcriptome of a cell contains different types of ncRNA that can be divided into two principal classes (Table 1): structural and regulatory ncRNAs. Structural ncRNAs include RNA molecules that are usually constitutively expressed such as ribosomal and transfer RNAs. Regulatory ncRNAs can be classified into three major classes based on transcript size: small (small ncRNAs), medium and long non-coding RNAs (lncRNAs) [6]. The most studied class of small ncRNAs in cardiovascular research is the microRNAs (miRNAs). MiRNAs are endogenous, single-stranded molecules consisting of approximately 20–22 nucleotides that regulate their target genes by reducing mRNA stability and/or translation [11]. Changes in microRNA expression lead to changes in gene function. This dysregulation of miRNA expression appears to play a significant role in the onset and progression of cardiovascular diseases [12]. Despite the progress in defining the role of microRNAs in cardiac and vascular biology, the complex network of ncRNAs and their interaction with different states of cardiovascular development and disease is still unknown. This is related to the multiple diversity of biogenesis, expression and functional properties of different classes of ncRNAs. Among these, the long non-coding RNAs (lncRNAs) are apparently the most numerous and functionally different [13]. LncRNAs are broadly classified as transcripts longer than 200 nucleotides and some of them are preferentially expressed in specific tissues [14]. Thus it is becoming increasingly clear that lncRNAs can regulate numerous molecular mechanisms. Recently, lncRNAs have emerged as new players in cardiovascular development and disease demonstrating potential roles in different cellular processes [15,16]. However, the characteristics and functions of the overwhelming majority of these lncRNAs are currently unknown. Accordingly, the functional characterization of lncRNAs is essential to advance our comprehensive understanding of cellular processes underlying cardiovascular development and disease. In the present review, emerging roles of ncRNAs in cardiovascular pathophysiology are discussed. Particular focus will be on the evaluation of biological roles of microRNAs and lncRNAs in vascular as well as cardiac disorders. Moreover, the focus of this review is to provide an overview of the current state of knowledge of molecular processes implicated in differentiation and cardiovascular development, which are related to the function of ncRNAs.

## 2. An Overview on the Main Methods to Analyze the ncRNAs Expression

Each ncRNA has expression levels that are tissue- or stage-specific. In recent years several methods have been developed to study ncRNA expression. A common approach is Real-time PCR, which is employed mainly to analyze microRNA expression levels but can be used also for studies on long ncRNA [17]. Also many approaches based on immunoprecipitation assays have been developed in recent years (e.g., RNA immunoprecipitation or RIP, Cross-linking and immunoprecipitation or CLIP, RNA-chromatin immunoprecipitation or RNA-ChIP) [18–20]. RNA-IP was developed to identify ncRNAs, especially ncRNA, that interact with a specific protein. The basic principal behind all immunoprecipitation approaches is the same. Using a specific antibody it is possible to isolate a ncRNA-protein complex, then a cDNA library is constructed and the ncRNA is sequenced. Unfortunately, for any of these immunoprecipitation-based approaches the results are influenced by the specificity and affinity of the antibodies. Moreover, these methods (Real-time PCR or IP) allow evaluation of the expression of a few specific molecules but do not permit the discovery of new ncRNAs or provide an overview of all ncRNAs. Recently, advances in technology enabled the development of new genome-wide screening methods to study ncRNAs and their targets. Among these the most commonly used are microarray analysis and RNA sequencing. These technologies are very accurate and permit large-scale analysis of ncRNAs. In particular, the microarray [21–23] approach offers various platforms allowing the study of microRNAs and mRNAs targets, although to date there are only a few chips to analyze long non-codingRNA. Analysis with traditional microarrays is limited to detecting the presence or the absence of known ncRNAs and it is incapable of identifying new molecules or revealing different splicing variants. To get around this problem a new approach has been defined: tiling arrays. Unlike traditional microarrays, these platforms permit identification of new ncRNAs in a selected DNA region without prior knowledge of their precise location. For instance, Rinn *et al*. used this approach to study lncRNAs expressed in the region of HOX genes in humans [24]. The RNA sequencing or RNAseq [25] refers to the use of high-throughput sequencing technologies to get information about a sample’s RNA content. This approach permits information to be obtained on differential expression of the interest gene, microRNA or long ncRNA. RNAseq is very sensitive in detecting less-abundant transcript and it can reveal alternatively spliced isoforms. Moreover, sequencing the entire transcriptome has been widely used to discover new non coding molecules. However, given the time and the cost related to the downstream analysis of the data generated by RNA sequencing, microarrays remain the first choice in many applications.

## 3. Functions of Non Coding RNAs

Although the function of most lncRNAs remains unknown, it has become clear that these molecules are intimately involved in many biological processes. LncRNAs can regulate gene expression programs through a variety of mechanisms, such as epigenetic modifications of DNA, alternative splicing, post-transcriptional gene regulation and mRNA stability and translation [5,26,27]. Given their established roles in transcriptional regulation, lncRNAs play a key role in several cellular events including proliferation, migration, apoptosis and development [21,28]. LncRNAs are now known to regulate the expression of protein-coding genes: they can positively or negatively control the expression of their target genes. Several lncRNAs are involved with *in cis* inactivation of larger genomic regions by epigenetic mechanisms. Kcnq1ot1 is a regulatory non coding antisense RNA that regulates epigenetic gene silencing in an imprinted gene cluster *in cis* [29]. This lncRNA specifically interacts with nearby genes in embryonic tissues causing transcriptional gene silencing. More recently, it was found that lncRNAs can act *in cis* to regulate expression of neighboring genes during cardiomyocyte differentiation [30]. Notably, many lncRNAs are now known to regulate the expression of genes by a *trans* mechanism. One example of a lncRNA that acts *in trans* is AK143260, termed Braveheart (Bvht) that specifically promotes activation of a core gene regulatory network to direct cardiovascular lineage commitment [15]. So far, several other functions have been attributed to lncRNAs. These molecules can act as scaffolds bringing together multiple proteins to form ribonucleoprotein complexes. For example, Miao-Chih Tsai *et al*. showed that a long non coding transcript, termed HOTAIR (HOX Antisense Intergenic RNA), acts as a scaffold for Polycomb Repressive Complex 2 (PRC2) and LSD1/CoREST/REST complex [31]. In addition to their role in chromatin regulation, lncRNAs can also function as molecular “decoys” of transcription factors and other regulatory proteins. PANDA (P21 associated ncRNA DNA damage activated) is an example of a lncRNA with decoy functionality. In fact, PANDA interacts with the transcription factor NF-YA to limit expression of pro-apoptotic genes [32]. Finally, the presence of a complex network of interactions between lncRNAs and miRNAs is becoming increasingly clear. In fact, lncRNAs may exert their biological activity through their ability to act as endogenous decoys for miRNAs. For example, a muscle-specific long noncoding RNA, linc-MD1, could interact with two specific miRNAs, miR-133 and miR-135, and promote muscle differentiation by acting as a competing endogenous RNA (ceRNA) in mouse and human myoblasts [33]. Another lncRNA which has been identified in association with microRNAs is the pseudogene PTENP1 [34]. Similar to Linc-MD1, PTENP1 mRNA acts as a decoy for miRNAs that directly target the tumor suppressor protein PTEN. Accordingly, PTENP1 reduces down-regulation of PTEN messenger RNA. Recent reports also show that stable circular lncRNAs (circRNAs) can act as molecular decoys of microRNAs [35,36]. Taken together, these observations suggest that lncRNAs could have profound effects on several molecular mechanisms. Nevertheless, lncRNAs are poorly conserved among species resulting in an additional degree of complexity in the definition of their functions. Despite rapid progress in lncRNA discovery, evidence of physiologic function for lncRNAs remains poor and further investigation is necessary.

## 4. Roles of ncRNAs in Vascular Biology and Disease

**Figure 1:**
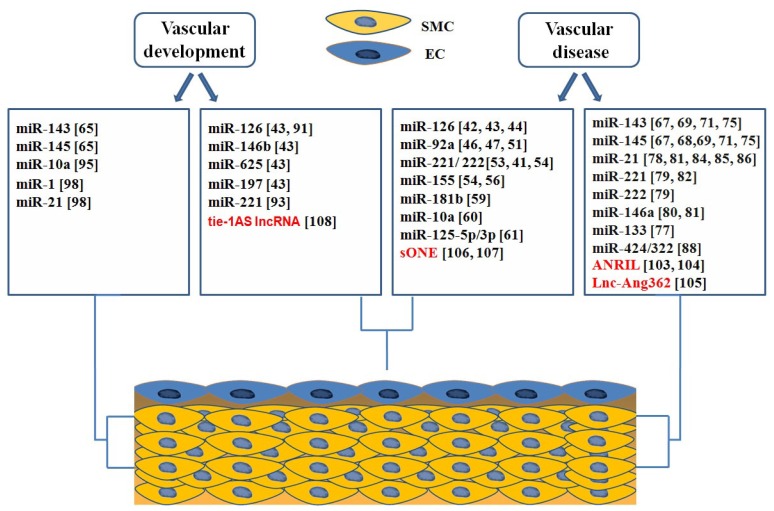
Role of non-coding RNAs in Vascular Development and Disease.

The vessel wall is composed of endothelial cells (ECs) and smooth muscle cells (SMCs) that play central roles in vascular biology and disease. In fact, these cells can undergo profound changes in phenotype during vascular injury and remodeling; these changes are correlated with pathologies such as atherosclerosis and proliferative thickening of the vessel known as restenosis. Atherosclerosis is a chronic inflammatory disease of the arterial wall and is the major cause of death in western countries [37]. It is a complex process involving multiple cell types and the interactions of many different molecular pathways. The events that lead to the formation of atherosclerotic lesions include modification of endothelial cell function, monocyte adherence and entry into vessel wall, phenotypic modulation of smooth muscle cell, and platelet adhesion and aggregation [38]. Phenotypic modulation of smooth muscle cells is, also, crucial in the neointimal lesion formation after stent implantation [39]. Numerous ncRNAs, especially microRNAs, have been shown to govern these processes during vascular disease. In fact, miRNA control endothelial cell and vascular smooth muscle cell biology, and thereby regulate the progression of vascular disease, such as atherosclerosis and restenosis. Current evidence also suggests that other ncRNA classes, such as lnc-RNA molecules play a critical role in endothelial and smooth muscle cell function. Figure 1 summarizes the role of ncRNA classes in different cells of the vessel wall.

### 4.1. microRNAs in Endothelial Biology and Dysfunction

In endothelial cells (ECs) the action of specific miRNAs is important for vascular signaling and function. Different studies indicate that the major miRNA-regulating enzymes, Dicer and Drosha, are essential for angiogenic functions of endothelial cells [40,41]. The endothelial-specific miR-126 is the most abundant miRNA found in adult ECs and it is involved in endothelial dysfunction and inflammation [42]. It is interesting to observe how miR-126 regulates the response of ECs to VEGF by inhibiting sprout-related protein SPRED1, a negative inhibitor of VEGF signaling [43]. Another group demonstrated that VCAM-1 is a direct target of miR-126 [44]. In the early phase of atherosclerotic disease, inflammatory cytokines increase a series of adhesion molecules, such as VCAM-1, on the surface of ECs. Inhibition of miR-126 increases leukocyte adherence in TNFα-stimulated ECs. Endothelial cell functions are critically regulated by other microRNAs: the miR-17-92 cluster, a polycistronic miRNA gene that produces six mature miRNAs: miR-17, miR-18a, miR-19a, miR-19b-1, miR-20a, and miR-92a [45]. Individual members of the miR-17-92 cluster, function as negative regulators of angiogenesis. In particular, miR-92a inhibited angiogenesis by targeting several functional genes, including integrin α5 (ITGa5) [46]. In addition, miR-92a negatively regulates KLF2 and KLF4 expression in athero-susceptible endothelium [47]. Given that both endothelial KLF4 and KLF2 are implicated in protection against atherogenesis [48–50], miR-92a may be important in arterial disease. Moreover, we have recently analyzed the effect of miR-92a in endothelial cell by loss-of-function studies [51]. Our group demonstrated that systemic administration of a complementary oligonucleotide (antagomiR-92a) significantly enhanced re-endothelialization in carotid arteries after balloon injury or arterial stenting. Our group and others [46,51] showed the relationship between miR-92a and endothelial nitric oxide synthase (eNOS) expression. Nitric oxide (NO) limits the formation of neointimal hyperplasia in animal models of arterial injury to a large part by inhibiting vascular smooth muscle cell proliferation [52]. Accordingly, the functional consequences of the miR-92a inhibition are an increase in NO bioavailability and an antiproliferative effect on SMCs [51]. A further example of negative correlation between microRNAs and eNOS activity is represented by miR-221 and miR-222. These microRNAs are highly expressed in ECs and exhibit anti-angiogenic effects [53]. Notably, over-expression of miR-221 and miR-222 indirectly reduces the expression of eNOS [33]. miR221 and miR-222 directly target c-kit, the receptor for stem cell factor (SCF), which plays a key role in endothelial cell migration [53]. Recently it has been shown that the miR-221 and miR-222 are negatively correlated with the expression of Ets-1 [54] that regulates the expression of several inflammatory molecules in the endothelial cell during vascular inflammation [55]. Another miRNA which has been identified in endothelial cells is miR-155. Similar to miR-221 and miR-222, miR-155 directly targets ETS-1 in ECs [54]. Also, miR-155 down-regulates eNOS expression through decreasing eNOS mRNA stability by binding its 3′-UTR [56]. Given their role in regulating endothelial cell biology, miR-221, miR 222 and miR-155 represent possible therapeutic targets in the inflammatory response of endothelial cells during the initial stage of atherosclerosis. Several other groups provide additional examples of the intersection between microRNAs and endothelial cell activation and dysfunction. In response to inflammatory stimuli, the nuclear factor-KappaB (NF-κB) signaling pathway is activated leading to the expression of multiple pro-inflammatory genes in ECs [57]. In fact, in Apolipoprotein E (ApoE)-deficient mice, endothelial cell-specific inhibition of NF-κB resulted in reduced development of atherosclerosis [58]. Two endothelial-specific microRNAs, miR-10a and miR-181b, inhibit the activation of the NF-κB signaling pathway in ECs. Recently, miR-181b has been identified as a key player in vascular inflammatory disease. miR-181b expression is reduced in response to TNF-a in the vascular endothelium, whereas its over-expression inhibits TNF-α-induced NF-κB-responsive targets gene such as VCAM-1 and E-selectin [59]. Moreover, miR-181b targets importin-α3, a critical protein in NF-κB nuclear translocation and activation. miR-10a directly inhibits mitogen-activated kinase kinase kinase 7 (MAP3K7) and beta-transducin repeat-containing gene (β-TRC) [60]. These molecules are essential in promoting IκBα degradation, an inhibitor of NF-κB activation. Inhibition of miR-10a enhances the NF-κB-dependent expression of adhesion molecules in ECs. Other specific microRNAs that regulate endothelial cell function have been described. For example, miR-125a-5p and miR-125b-5p have been identified as negative regulators of ET-1 [61], a potent vasoconstrictive and mitogen peptide that plays multiple roles in the progression of vascular disorder [62]. Taken together, the results described above indicate that several microRNAs play an essential role in endothelial pathophysiology. Accordingly, the identification of specific microRNAs involved in biological processes, such as angiogenesis and inflammation, could lead to the definition of new strategies to treat vascular diseases. Given that the same microRNAs may have opposite effects in different biological contexts, further studies are necessary to clarify their roles in endothelial dysfunction. For example, identification of signaling pathways which modulate the activity of microRNAs is critical for development of microRNA-based therapeutic strategies.

### 4.2. microRNAs in Phenotypic Switching of VSMCs

SMCs within adult animals retain remarkable plasticity and can undergo profound and reversible changes in phenotype, a process referred to as phenotypic switching [63]. SMCs play a role during all phases of the atherogenic process as well as in proliferative disease [64–66]. Several microRNAs are implicated in VSMC phenotypic switching in response to vascular injury or atherosclerotic disease.

#### 4.2.1. miR-143 and miR-145 Play a Role in the Regulation of Phenotype of VSMCs in Response to Injury

miRNA-143 and -145 are considered the master regulators of contractile phenotype by promoting contractile protein expression [67]. The expression levels of miR-143/145 are down-regulated in injured or atherosclerotic vessels and are associated with the phenotypic switch from a contractile/quiescent to a synthetic/proliferative phenotype. A recent study reported that adenovirus-mediated over-expression of miR-145 could partially restore down-regulation of SMC marker genes and neointima formation following balloon injury of the rat carotid artery [68]. In addition, miR-143/145-knockout mice present morphological changes in the aorta, due to an incomplete differentiation of VSMCs [69]. Several growth factors promote phenotypic switching of VSMCs during vascular disease [63]. The growth factor, PDGF-BB, is a critical regulator of VSMCs phenotype in vessel injury. Indeed, PDGF stimulation increases migration and proliferation of SMCs *in vitro* and *in vivo* [70]. It has been shown that PDGF can reduce miR-145 and miR-143 expression through Src and p53 activity [71] and promote formation of podosomes. miR-143 and miR-145 directly target key regulators of podosome formation, such as PDGF receptor α (PDGF-Rα), protein kinase Cɛ (PKCɛ) and fascin. Another cytochines that participate in the phenotypic control of VSMCs are Transforming Growth Factor beta (TGFβ) and bone morphogenetic proteins (BMPs). Unlike PDGF, the TGF-family of growth factors has been shown to promote contractile phenotype [72–74]. Induction of miR-143 and miR-145 by TGF-β or BMP4 leads to down-regulation of KLF4 expression and activation of contractile genes [75]. It is interesting to note that the expression of miR-143 and miR-145 is regulated by multiple growth factor signaling pathways promoting phenotypic modulation of SMC. Therefore, because miR-143 and mir-145 may be important modulators of vascular disorder, further studies are necessary to define the regulatory mechanisms of their expression in response to vascular injury or atherosclerotic disease.

#### 4.2.2. miR-133 Is a Negative Regulator of SP-1 and Promotes Contractile Phenotype of VSMCs

Similarly to miR-143 and miR-145, a number of additional microRNAs play a role in smooth muscle cell phenotypic switching and in vascular disease. In a recent study from our laboratory, we found that miR-133 has a potent inhibitory role on VSMC phenotypic switching. miR-133 specifically suppresses Sp-1 expression *in vitro* and *in vivo* and participates in a complex network with Serum response Factor (SRF) in regulating smooth muscle gene expression. Sp-1 is a key regulator of KLF4 expression in phenotypically modulated SMCs [76]. KLF-4 is up-regulated in VSMCs following vascular injury and inhibits myocardin-induced SMC marker gene expression. Following balloon injury of the rat carotid artery there is an increase of Sp1 expression in the neointima related to the acquisition of proliferative/synthetic phenotype of VSMCs. Accordingly, over-expression of miR-133 reduces neointima formation and SMCs proliferation after balloon injury of the rat carotid artery [77].

#### 4.2.3. microRNAs that Promote De-Differentiated Phenotype of VSMCs

Other miRNAs have been implicated in the phenotypic modulation of SMCs: miR-21, miR-146a, miR-221, and miR-222. Unlike the miR-143/145 cluster and miR-133, these microRNAs were found to be significantly up-regulated after vascular injury [78–80] and their inhibition reduces neointimal formation following balloon injury of rat carotid arteries *in vivo*. Therefore, expression profiles of microRNAs in human atherosclerotic plaques in comparison to control, demonstrate that miR-21 and miR-146a were up-regulated in human atherosclerotic plaques, whereas several predicted targets of these miRNAs were down-regulated [81]. miR-221 and miR-222 contribute to SMC phenotype by repressing specific targets such as p57Kip2 and p27Kip1 [79]. They are cyclin-dependent kinase inhibitors and have an antiproliferative effect on VSMCs. Interestingly, PDGF induces the expression of miR-221, leading to down-regulation of multiple target genes and promoting the proliferation of SMCs [82]. Accordingly, inhibition of miR-221 prevents reduction of p27Kip1 in response to PDGF, as well as VSMC proliferation. miR-146a promotes SMCs proliferation *in vitro* and neointimal hyperplasia *in vivo* [80]. Notably, miR-146a inhibits KLF4 expression by targeting its 3′-UTR. Inhibition of miR-146a increases KLF4 expression, while its over-expression induces an opposite effect. It has been reported that KLF4 has a critical role in the regulation of the SMCs phenotype [83] and its expression is correlated to different microRNAs. Particularly, miR-143 and miR-145 directly target KLF4 in SMCs [75]. miR-146a promotes the proliferative phenotype of SMCs through a reduction of KLF4 expression; in contrast, KLF4 regulation of miR-145 and miR-143 promote the contractile phenotype of SMCs. Thus, further studies are necessary to define the regulatory mechanisms of KLF4 in SMCs. miR-21 promotes the cellular response that leads to proliferative thickening of the vessel by directly targeting Phosphatase and Tensin Homolog (PTEN), a critical regulator of SMCs function both *in vivo* and *in vitro* [78]. SRF is a transcription factor that plays a critical role in SMCs biology, influencing both proliferation and differentiation depending on the types of coactivators or repressors present at specific cellular stages. A recent report demonstrated that Serum Response Factor (SRF) regulates PTEN expression through a reduction of miR-21 levels [84]. Regulation SRF-mediated of miR-21 occurs through a miR-143-dependent signaling pathway. Recently, the role of miR-21 in the regulation of SMC phenotype was correlated with abdominal aortic aneurysm (AAA). The expression levels of miR-21 increase during development of AAA in two murine models [85]; moreover Lentivirus-mediated over-expression of miR-21 induced cell proliferation of SMCs, with protective effects on aneurysm expansion. In addition, miR-21 targets several signal molecules associated with SMCs phenotype such as Programmed Cell Death 4 (PDCD4) [86], B-cell leukemia/lymphoma 2 (BCL-2) [78], and Tropomyosin 1 (TPM1) [87]. These observations indicate that miR-21 could play important roles in diverse vascular diseases. Different microRNAs can be involved in the same process; for example recent studies [88] demonstrated that miR-424/322 also play a key role in modulation of SMCs phenotype in response to vascular injury. In particular, ectopic expression of miR-424/322 induces inhibition of proliferation and migration in SMCs and reduces restenosis in injured carotid arteries in rats. miR-424/322 regulates SMCs phenotype suppressing its direct targets cyclin D1 and CA+2 regulating proteins (calumenin). Interestingly, miR-424/322 is significantly up-regulated after vascular injury and this suggest that miR-424/322 is correlated to an adaptive response to counteract proliferation of SMCs. To summarize, these findings suggest a new therapeutic strategy for vascular diseases connected with phenotypic switching of SMCs. Interestingly, chemically modified antisense oligonucleotides, termed “antagomirs”, have been used to decrease miRNA expression and function in different animal models [32,37,64]. Given that many microRNAs are increased after injury, it is possible that their specific inhibition by antagomirs could be considered as potential therapeutic targets for several vascular diseases. Nevertheless, further research is needed regarding the role of these aberrantly expressed microRNAs in SMCs.

### 4.3. Roles of microRNAs in Vascular Development

The formation of a vascular system requires the creation and remodeling of a continuous series of vessels. They are made mainly by endothelial cells, but smooth muscle cells ensure the correct tone and contractility of the vessels necessary for proper blood flow. After birth, VSMCs retain remarkable plasticity; they can switch between a contractile and proliferative phenotype, a characteristic fundamental in vascular development and remodeling. Both these processes are regulated by numerous factors, including microRNAs. miRNAs have been implicated in endothelial cell differentiation and are involved in the regulation of formation of blood vessels during vascular development. Knockout of DICER, the enzyme responsible for the maturation of microRNAs, reduces postnatal angiogenesis in response to several stimuli, such as exogenous VEGF, tumors, limb ischemia, and wound healing [89]. Specific silencing Dicer using siRNA, increases activation of the eNOS pathway but reduces proliferation and cord formation of human endothelial cell *in vitro* [41].

#### 4.3.1. miRNAs Involved in Endothelial Development

The first miR shown to be essential for vessel formation and integrity is miR-126. It is a positive regulator of angiogenic signaling in endothelial cells and also of vascular integrity *in vivo*. miR-126-deficient endothelial cells failed to respond to various angiogenic factors, including VEGF, EGF and bFGF [43,90,91]. Studies on zebrafish show that down-regulation of this microRNA reduces vascular integrity and induces hemorrhages [43]; furthermore, studies in mice demonstrate that the deletion of miR-126 causes defects in endothelial cell proliferation, migration and angiogenesis [91]. However, both these studies demonstrate that miR-126 affects endothelial cell function but it is not essential for cell differentiation or embryonic vessel formation. Fish *et al*. show additionally that miR-146b, miR-625 and miR-197 appear up-regulated in mouse ESC-derived endothelial cells, however the role of these miRNAs in the vascular system is still unknown [43]. Kane *et al*., in order to study the endothelial differentiation, carried out a Taqman low-density array (TLDA) analysis of miRNA levels in pluripotent hESCs and in hESCs differentiated for 10 days. They found that up-regulation of miR-126 causes an increase in levels of further microRNAs such as let-7 family, miR-210, miR-130a, miR-196, miR-133a [92]. However, despite the fact that all of these microRNAs are modulated during endothelial differentiation, not one of them has been shown to be directly correlated to endothelial cell control. Recently, Nicoli *et al*. investigated the role of miR-221 in endothelial cells in vascular development; they demonstrated that miR-221 promotes endothelial cell proliferation and migration through repression of two targets: cyclin dependent kinase inhibitor 1b (cdkn1b) and phosphoinositide-3-kinase regulatory subunit 1 (pik3r1). Also miR-221 expression is inhibited by Notch signaling [93].

#### 4.3.2. microRNAs in Regulation of Vascular Smooth Muscle Cell Differentiation

Several studies have provided compelling evidence that microRNAs play a critical role in the initial specification of vascular smooth muscle cell lineage during development. Indeed, inactivation of Dicer in VSMCs results in late embryonic lethality due to decreased VSMC proliferation and differentiation and due to vascular abnormalities and extensive hemorrhage [94]. Thus, the function of Dicer-generated miRNAs is essential during development of VSMC. A recent report demonstrated a crucial role for miR-143 and miR-145 in VSMC differentiation [65]. The down-regulation of miR-145 using cholesterol-modified antisense oligonucleotides inhibits myocardin-induced reprogramming of fibroblasts into SMC and represses expression of multiple SMC markers, such as ACTA2, MyH11 and Calponin. Also, miR-145 over-expression is sufficient to induce differentiation of multipotent neural crest stem cells into smooth muscle cells and to inhibit their proliferation. Accordingly, miR-143 and miR-145 target a network of factors, such as KLF4 and ELK-1, in order to promote VSMCs differentiation and repress proliferation. Other miRNAs that may promote VSMC differentiation are miR-10a, miR-1 and miR-21. Recently, it has been shown that miR-10a is involved in the differentiation to smooth muscle cell lineage from mouse ESCs in response to Retinoid acid [95]. Several studies indicate that Retinoid Signaling positively influences the SMC differentiation program from stem cells [96]. Notably, miR-10a is up-regulated during retinoid acid-induced SMC differentiation. Furthermore, miR-10a directly targets histone deacetylase 4 (HDAC4), which is a negative regulator of SMC differentiation [97]. miR-1 plays a critical role in the SMC lineage differentiation in embryonic stem cell-derived SMC cultures[98]. miR-1 expression is highly up-regulated during differentiation of mouse embryonic stem cell (ESC) to SMCs. miR-1 has been implicated in SMC differentiation by directly targeting the 3’UTR of KLF4 and enhancing expression of the smooth muscle-restricted markers gene. miR-21 was shown to promote differentiation of VSMCs in response to transforming growth factor and bone morphogenetic protein stimulation [99]. miR-21 directly targets PDCD4 (programmed cell death 4), which acts as a negative regulator of smooth muscle contractile genes.

### 4.4. Long Non Coding RNAs in Vascular Development and Disease

Although the heterogeneous group of lncRNAs play a wide range of roles in cellular function, their characterization pertaining to vascular development and disease is limited to only a few examples. Variation on chromosome 9p21 is associated with risk of coronary artery disease (CAD) [100,101]. This genomic region contains a long intergenic noncoding RNA, designated antisense noncoding RNA in the INK4 locus (ANRIL). ANRIL is a long non-coding RNA which is transcribed from the INK/ARF locus. ANRIL is expressed in tissues and cell types that are affected by atherosclerosis such as primary coronary smooth muscle cells, vascular endothelial cells, human monocyte-derived macrophage cells and RNA extracted from carotid and arterectomy [102]. Notably, increased expression of ANRIL transcripts was directly correlated with the severity of atherosclerosis [103]. However, despite the potential importance of ANRIL to vascular disease, the pathophysiology underlying the link between ANRIL and coronary artery disease remains currently unknown. ANRIL has been associated with epigenetic silencing of the INK4B-ARF-INK4A locus on chromosome 9p21.3 [104]. In fact, ANRIL binds the p15(INK4b) transcript and recruits the Polycomb Repressor Complex (PRC) to repress the transcription of genes at this locus. Therefore, it is possible that the increased expression of ANRIL is correlated with altered expression of p15INK4B leading to coronary artery disease. Future studies will be necessary to define the role of ANRIL in vascular disease. Using whole transcriptome sequencing, a recent publication revealed the expression profile of lncRNAs in VSMCs in response to Ang II [105]. In this paper, the authors showed that two miRNAs, miR-221 and miR-222, are co-transcribed with a specific lnc-RNA, Lnc-Ang362. These microRNAs have been found to play a critical role in smooth muscle cell proliferation and neointimal hyperplasia in response to vascular injury [79,82]. Interestingly, knockdown of Lnc-Ang362 reduces the expression of these miRNAs as well as cell growth. Correlations between the expression of lncRNAs and miRNAs raise the intriguing possibility of complex functional regulatory pathways in which several types of ncRNAs interact and influence the phenotype of VSMCs during vascular disease. Future studies are needed to dissect the exact roles of lncRNAs in phenotypic switching of VSMCs. A further example of a ncRNA correlated with vascular disease is a natural antisense transcript (NAT), termed sONE. A key function of this lncRNAs is the regulation of eNOS expression in a post-transcriptional manner under normoxic and hypoxic conditions [106,107]. Over-expression of sONE in endothelial cells reduces eNOS expression. Alterations of NO production by the vascular endothelium results in endothelial dysfunction, which occurs as a prelude to atherosclerosis. Thus it could be interesting to investigate the role of antisense lncRNA sONE in the post-transcriptional regulation of eNOS in order to define a potential therapeutic target in vascular disease. More recently, Keguo Li *et al*. showed that a new long noncoding antisense transcript, termed tie-1AS lncRNA, is required for the regulation of tyrosine kinase containing immunoglobulin and epidermal growth factor homology domain-1 gene (tie-1) levels *in vivo* and *in vitro* [108]. Analysis of tie-1AS lncRNA and tie-1 revealed that the ratio of tie-1 versus tie-1AS lncRNA is opposite in normal placenta tissue compared with vascular anomaly tissue. Also, the tie-1AS lncRNA selectively binds tie-1 mRNA, resulting in down-regulation of tie-1 protein and thus specific defects in endothelial cell contact junctions. Over-expression of tie-1 AS lncRNAs resulted in defects in endothelial cell junctions and tube formation. For the first time these results identified a lncRNA that plays a functional regulatory role with potential implications in the control of vascular development. In summary, the results described above indicate that lncRNAs are involved in different aspects of development and disease but their role in the cardiovascular system remains to be further investigated.

## 5. ncRNA in Heart Development and Pathophysiology

The heart is the first organ to form during embryo development, a complex process involving many classes of regulatory molecules. Heart uninterrupted contractility and correct function are essential for life; its alterations are associated with numerous diseases, including atherosclerosis and stroke [7]. Due to its importance, a complex system of transcription factors closely intertwined with a family of different molecules precisely controls multiple aspects of heart development, function and dysfunction. In these mechanisms, a central role is played by ncRNAs.

### 5.1. microRNA in Heart Development

#### 5.1.1. miRNAs Encoded by MHC Genes

Two miRNAs highly expressed in the heart, miR-1 and miR-133, are implicated in the control of cardiac growth, regulating fundamental aspects of heart development *in vivo*. There are two isoforms of miR-1, miR-1-1 and miR-1-2, whereas miR-133 presents three isoforms, miR-133a-1, miR-133a-2 and miR-133b. These miRNAs are strictly related, in fact miR-1 and miR-133a are encoded by the same gene, in particular miR-1-1/miR-133a-2 and miR-1-2/miR133a-1. The expression of both these clusters is under the control of SRF and Mef2. SRF enhances the expression of these miRNAs in ventricular and atrial myocytes through a serum response element. Instead, Mef2 binds an intronic enhancer of these miRNAs to activate their expression in ventricular myocytes [109]. During differentiation from ES cells, miR-1 and miR-133 are expressed to promote mesoderm induction and to suppress the differentiation in other lineages, in mice [110,111]. Furthermore, the over-expression of miR-133 causes the inhibition of cardiomyocyte proliferation. Particularly, the up-regulation of miR-133 results in embryonic lethality due to the thinning of the ventricular walls and VSDs (Ventricular Septal Defects); whereas miR-133a-null mice present an ectopic expression of smooth muscle genes in the developing heart as well as aberrant cardiomyocyte proliferation [112]. The up-regulation of miR-1 causes embryonic lethality due to a deficiency in cardiomyocytes [109]. Furthermore deficient miR-1 mice exhibit an increased number of proliferating cardiomyocytes [113]. Other microRNAs encoded by MHC genes are implicated in heart development and stress responsiveness. α-MHC encodes for miR-208a, a heart-specific miRNA, while a closely related microRNA, miR-208b, is encoded by β-MHC. Both these miRs have the same seed sequence, but differ in their 3’UTR region. miR-208a is highly expressed in adult mouse heart, whereas mir-208b is very abundant in embryonic heart but it is present at low levels in an adult heart. Furthermore, while miR-208b is expressed in the heart and in other tissues like skeletal muscle, miR-208a is only expressed in the heart [114]. Interestingly the expression of MHC genes, essential for cardiac muscle contraction, is not equal during life; in fact α-MHC is the predominant myosin isoform in the adult heart, whereas β-MHC is highly expressed in the developing heart but is down-regulated after birth. Cardiac stress and diseases modulate MHC gene transcription, causing a switch in myosin content in the heart, which has a marked effect on cardiac contractility and function [115]. Clearly miR-208a and miR-208b follow the trend of their host genes, hinting that they have a role in the regulation of the α-MHC to β-MHC switch and consequently in cardiac conduction, in arrhythmias and in other aspects of the stress response. Nevertheless none of these microRNAs are essential for function of the adult heart, but they appear to function primarily to adapt adult cardiac gene expression to physiological and pathological signaling.

#### 5.1.2. microRNAs in Heart Chamber Morphogenesis

The heart is composed of cells with similar origin that develop divergent patterns of gene expression with numerous transcriptional networks that establish chamber or domain-specific gene expression and function. A precise regulation in time and space of gene expression and protein activity is necessary for a correct cardiac patterning. The most important microRNA in this process is miR-138; it was studied in zebrafish which represents an excellent model to study heart development, despite it having a heart containing a single atrium and ventricle [116]. Morton *et al*. show that miR-138 is required for cardiac maturation, in fact knock-down of this miR in zebrafish embryo causes the failure of ventricular cardiomyocytes to fully mature. So, miR-138 is necessary to establish an appropriate chamber-specific gene expression pattern during embryo development [116]. This miRNA is expressed in specific domains of the heart and targets various members of different pathways, particularly of retinoic acid (RA). It establishes discrete temporal and spatial domains of gene expression during cardiac morphogenesis, ensuring a correct development of the heart. Another microRNA required for proper morphogenesis of heart chambers is miR-143. It directly targets adducin3 (add3), an F-actin capping protein. As reviewed by Taber [117], alterations in cytoskeletal dynamics could drive cardiac morphogenesis by promoting regional changes in cell size and shape. So, the regulation operated by miR-143 on add3-pathway modulates cytoskeletal protein and, consequently, it could influence heart development and particularly chamber formation through active adjustment of myocardial cell morphology [118].

#### 5.1.3. microRNAs in Valves Development

**Figure 2:**
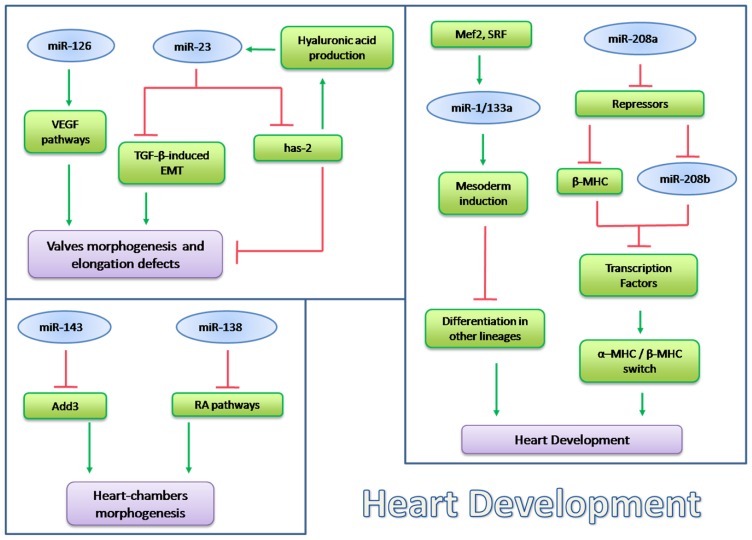
miRNAs involved in Heart Development. Schematic representation of the relevant microRNAs involved in heart development with a subset of their principal targets: miRNAs which regulate heart-chambers morphogenesis (miR-143, miR-138); miRNAs involved in valves morphogenesis and elongation defects (miR-126, miR-23); miRNAs regulating heart differentiation and development.

Defects in cardiac valves are the most common subtype of cardiovascular malformation and, in adults, are a major cause of morbidity and mortality. One gene was found which was involved in the regulation of this process: miR-23. Lagendijk *et al*. studied miR-23 in endothelial cells of mice and demonstrated that in this model miR-23 was able to inhibit a TGF-β-induced endothelial to mesenchymal transition (EMT), a process that normally occurs during heart valve development. They proposed that miR-23, has-2 (hyaluronic acid synthase 2) and hyaluronic acid (HA) create a regulatory feedback loop that could respond to various signals, including TGF-β [119]. Recently it has been shown in zebrafish that the loss of this microRNA causes endocardial defects, including cushion formation. miR-23 acts by the down-regulation of has-2, an extracellular remodeling enzyme required for endocardial cushion and valve formation. So this microRNA in the embryonic heart is required to restrict endocardial cushion formation by inhibiting has-2 expression and extracellular hyaluronic acid production [119,120]. A recent study shows that miR-126 is also implicated in valve elongation defects [121]. As shown above, this miR has a role in VEGF signaling in the development of endocardial cells. miR-126 targets a subunit of PI3K and Spred1, two negative regulators of VEGF pathway, so it positively regulates VEGF signaling in heart valve morphogenesis [122]. Despite current knowledge on microRNA functions in cardiovascular development, our complete understanding of their role is far from complete. Figure 2 summarizes the role of microRNAs in heart development.

### 5.2. microRNA in Heart Pathophysiology

The possibility that microRNAs might participate in heart disease was first suggested by the discovery of distinctive patterns of microRNA expression in the hearts of normal mice *vs*. mice that suffered from heart disease [123]. Recent studies on miRNA expression in heart have identified a subset of miRNAs highly expressed in the normal heart and modulated during cardiovascular disease [124].

#### 5.2.1. miR-195 and miR-98/let-7b in Cardiac Hypertrophy

The first-characterized miRNA involved in inducing hypertrophic growth in the adult heart was miR-195. Adenoviral-mediated over-expression of this microRNA leads to dilated cardiomyopathy and heart dysfunction *in vivo*, it is also sufficient to induce hypertrophy in neonatal rat cardiomyocytes [123]. Chen and colleagues demonstrated the requirement of proper LKB1/STRAD/MO25 complex formation for full activation of AMPK signaling; miR-195 is sufficient to suppress MO25 expression and downstream targets of the LKB1/STRAD/MO25 pathway [125]. They hypothesized that miR-195 targets the LKB1/AMPK signaling axis in hypertrophic cardiomyopathy progression, implicating a functional role of this microRNA in this process.

Conversely, miR-98/let-7b has been demonstrated to mediate the anti-hypertrophic effect of thioredoxin (Trx1), an ubiquitously expressed antioxidant that inhibits NF-κB (nuclear factor kappa-light-chain enhancher of activated B cells), Ras and ASK1 (apoptosis signal-regulating kinase 1). Trx1 negatively regulates the protein kinase cascade known to stimulate hypertrophy. Particularly, Yang *et al*. studied the effects of miR-98 up-regulation or down-regulation on cardiac hypertrophy *in vivo*, at baseline and in response to Ang-II. These studies show that Trx1 negatively regulates Ang-II-induced cardiac hypertrophy through up-regulation of miR-98/let-7b but does not affect heart morphology at baseline [126]. A validated target of miR-98/let-7 is cyclin D2, a cyclin that plays a key role in hypertrophy mediated by this microRNA; the down-regulation of Trx1 causes the up-regulation of miR-98 and the inhibition of hypertrophy.

#### 5.2.2. miR-1 in Heart Physiopathology

The most abundantly expressed microRNA in human heart is miR-1; as described before it is clear that it has a key role in the developing heart. After subjecting the heart of mice to increased pressure overload a down-regulation of miR-1 resulting in an increase in cardiac mass and contractile dysfunction was observed [113,127]. Furthermore, miR-1 is down-regulated in several models of cardiac hypertrophy and heart failure, conversely its over-expression attenuates cardiomyocyte hypertrophy indicating that miR-1 down-regulation has a causative role in the pathogenesis of this disease [113]. It is interesting to note that miR-1 was shown to regulate different pathways implicated in heart hypertrophy. First, it modulates calmodulin and Mef2a, two mediators of calcium signaling, and in addition the transcriptional effectors MEF2A and GATA4, suggesting that miR-1 controls calcium signaling by different modalities simultaneously [113,128]. Second, a dysregulation of insulin-like growth factor (IGF-1) has also been involved in pathological hypertrophy; it is a validated target of miR-1. In exercised trained rats and cardiac-specific Akt transgenic mice, which are models of physiological cardiac hypertrophy, miR-1, as well as miR-133, are down-regulated [110]. There is an inverse correlation between miR-1 and IGF-1: the microRNA controls the expression of IGF-1 and IGF-1 receptor and reciprocally it is down-regulated by IGF-1 stimulation depending on the activation of PI3K/AKT pathway and repression of Foxo3 transcription factor. Accordingly, acromegalic patients, in whom there is an atypical synthesis of IGF-1, display increased cardiac mass and wall thickness [127]. Finally, miR-1 targets twifilin 1 (Twf1), a cytoskeletal regulatory protein that binds to actin monomers preventing their assembly into filaments. The level of Twf1 is inversely correlated with expression of miR-1, so it is expressed at low levels in an adult heart. Moreover down-regulation of miR-1 induced by hypertrophic stimuli results in increased Twf1 expression; likewise Twf1 over-expression is sufficient to induce cardiac hypertrophy in neonatal rat cardiomyocytes, suggesting the therapeutic relevance of modulation of Twf1 expression in attenuating cardiac hypertrophy [129]. Moreover, miR-1 was studied in a model of acute ischemic heart disease (IHD): in cardiomyocytes from ischemia/reperfused (I/R) rats, this microRNA appears up-regulated and inversely correlated with the anti-apoptotic protein Bcl-2 [130], suggesting a potential role of this miRNA in cardiomyocyte apoptosis. In heart failure models levels of miR-1, like miR-133, appear decreased; the same effect is observed in the hearts of patients with hypertrophic cardiomyopathy or atrial dilation [110,131]. The down-regulation of miR-1, and likewise that of miR-133, is associated with the increased levels of two members of the HCN ion channel family, HCN2/HCN4, in hypertrophic hearts. Probably the up-regulation of these channels may contribute to enhanced automaticity and arrhythmias in heart failure [131]. Moreover, a recent study demonstrated that miR-1 directly targets connexin 43 (Cx43), the main cardiac connexin, which has an aberrant increased expression in hypertrophic cardiomyocytes *in vitro* and *in vivo* [132]. Given the numerous processes regulated by miR-1, it could be an important therapeutic target, however alterations of miR-1 levels could alter several mechanisms, so other studies are necessary before its application in the medical field.

#### 5.2.3. miRNA Implicated in Calicineurin/NFATs Pathway

Cellular and *in vivo* models of cardiac hypertrophy induced by transverse aortic constriction and phenylephrine (PE) treatment involve increased activity and expression of calcineurin and decreased expression of miR-133 [133]. NATFc4, a member of calcineurin-activated NFAT family, has two functional binding sites for miR-133. Gain-of-function approaches show that miR-133 decreases NFAT mRNA levels as well as the hypertrophic response to PE-mediated stimulation in primary cardiomyocytes, and miR-133 loss-of-function increases NFATc4 expression and a hypertrophic response [123,134]. Moreover this microRNA decreases cardiac hypertrophy targeting RhoA, Cdc42 and Nelf-A/WHSC2 [110]. Various studies on this microRNA show that the over-expression of miR-133 attenuates agonist-induced hypertrophy [135]; conversely silencing of miR-133 makes the myocardium more sensitive to excessive cardiac growth [136]. Another member of NFAT family, NFATc3, is positively regulated by Myocardin, a transcriptional co-activator that promotes cardiac hypertrophy [137]. Under physiological conditions Myocardin is expressed at low levels but upon hypertrophic stimulation its expression is increased and consequently NFATc3 is up-regulated. Myocardin is a direct target of miR-9, so it was studied like a potential regulator of cardiac hypertrophy. Studies of miR-9 over-expression or inhibition, under hypertrophic stimulation, demonstrate that this microRNA negatively regulates cardiac hypertrophy, *in vivo* and *in vitro*, by targeting Myocardin [137]. Furthermore NFATC3 positively regulates miR-23a, which is up-regulated at transcriptional level by this factor. In fact, the expression of miR-23a is required to mediate hypertrophic growth in response to activation of the calcineurin/NFAT pathway; it directly targets an anti-hypertrophic protein: the muscle-specific ring finger protein 1 MuRF1 [134,138]. Another microRNA implicated in this process is miR-199b: it is a direct target of calcineurin-NFAT signaling, with an increased expression in heart failure. This microRNA modulates calcineurin-NFAT signaling-mediated hypertrophy in a positive feedback loop. Calcineurin induces miR-199b expression through a functional NFAT site upstream of the miR’s gene; then the miR targets Dyrk1a, the dual-specificity tyrosine (Y) phosphorylation-regulated kinase 1a, in a process that constitutes a pathogenic feed-forward mechanism affecting calcineurin-responsive gene expression. Mice over-expressing miR-199b exhibit a strong hypertrophic phenotype induced by calcineurin/NFAT signaling, whereas inhibition of miR-199b normalizes Dyrk1a expression, reduces nuclear NFAT activity, and inhibits and even reverses the cardiac hypertrophy and fibrosis in mice models of heart failure [139].

#### 5.2.4. miRNAs Regulated by Thyroid Hormone

miR-208a and miR-499 are implicated in the regulation of myosin gene expression and cardiac stress response. They play a redundant role: *in vivo* deletion of miR-208a resulting in viable animals with normal cardiac size at baseline, but these animals show a decline in cardiac function up to five months of age [140]. However miR-208a is under the thyroid hormone receptor TR, and its over-expression induces hypertrophic growth in mice by suppressing two negative regulators: thyroid hormone-associated protein-1 (Thap1) and myostatin [141]. Consistently elevated levels of miR-499 led to cardiomyopathy and cardiac hypertrophy in a dose-dependent manner [142]. miR-208a is up-regulated in response to a hemodynamic pressure overload and in heart failure [141]. It is a positive regulator of β-MHC, required for the development of cardiac hypertrophy and myocardial fibrosis [140].

#### 5.2.5. miRNAs Regulated by TGF-β in Heart Physiopathology

Fibroblasts are the most abundant class of non-cardiomyocyte cells in the heart; they produce ECM proteins as well as paracrine factors that can regulate the function of cardiomyocytes. In particular, in response to TGF-β, fibroblasts produce ECM and reduce collagenase production, leading to an excessive matrix accumulation. In this way, a key role is played by miRNAs regulated by TGF-β, a known agonist in the production and deposition of collagens in the heart, which contribute to cardiac hypertrophy. Numerous miRNAs are dysregulated in excessive fibrosis; including miR-29 and miR-21 [143]. The miR-29 family is composed of three members, 29a-b and -c, which are preferentially expressed in fibroblasts as compared with cardiomyocytes. All miR-29 family members target mRNA encoding multiple collagens, fibrillins and elastins and another multitude of ECM-related proteins involved in fibrosis. Interestingly, these microRNAs are down-regulated after TGF-β stimulation in cardiac fibroblasts, suggesting that they could contribute to TGF-β-induced fibrosis [129]. Another microRNA induced by TGF-β and dysregulated in fibroblasts, including in cardiac fibroblasts, in multiple types of stress, is miR-21 [144,145]. Studies on miR-21 reveal that this microRNA contributes to myocardial remodeling through regulation of ERK-MAPK-signaling, which is crucial in fibroblast survival and activation. The over-expression of miR-21 indirectly enhances the activity of ERK-MAP kinase; in fact it targets directly Sprouty-1 (SPRY1), a negative regulator of this pathway. In this manner miR-21 positively regulates cardiac fibroblast survival and growth factor secretion that eventually controls interstitial fibrosis and cardiac hypertrophy [143]. However there is a disagreement in the literature on the role of this microRNA: miR-21, in fact, induces the expression of matrix metalloproteinase-2 by targeting the phosphatase and tension homolog (PTEN) in fibroblasts [146]. Furthermore miR-21-null mice display fibrosis levels comparable to wild-type littermates, suggesting that this microRNA is not essential for pathological cardiac remodeling [147]. miR-21 has a potential role in the regulation of different mechanism for the decrease in cardiac contractile function in heart failure. Studies in tumor cells demonstrate that it targets the tropomyosin I (TPM1) [148], but it has not yet been studied in human heart failure.

#### 5.2.6. The Role of miR-378 in MAPK Signaling

**Figure 3:**
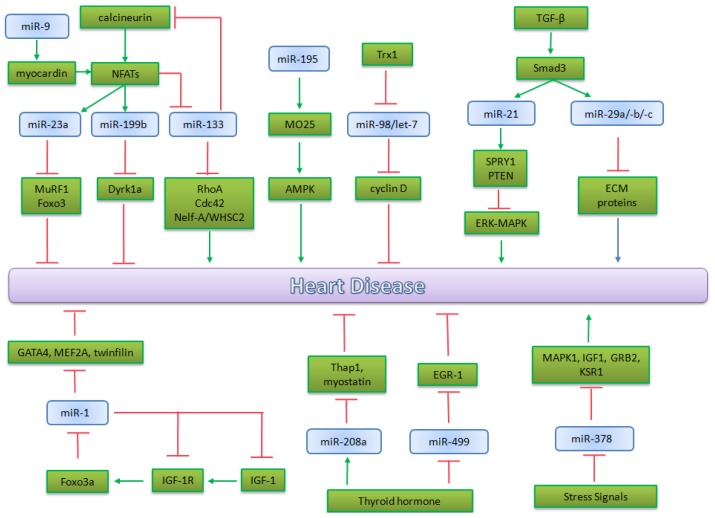
miRNAs involved in Heart Pathophysiology. A schematic overview of the relevant microRNAs implicated in principal heart disease (*i.e*., cardiac hypertrophy, heart failure and arrhythmias).

MAPK signaling is also controlled by miR-378. This microRNA is sufficient to repress cardiomyocyte hypertrophy regulating this pathway by targeting four members: MAPK1 itself, IGF-1, GRB2 and KSR1. Ganesan *et al*. studied miR-378 *in vivo* in a mouse model for chronic pressure overload (TAC); they demonstrate that the restoration of this microRNA in mice in which it was down-regulated, partially prevents cardiomyocyte hypertrophy and also does not trigger apoptosis *in vivo* [149]. So the tissue-specific up-regulation of miR-378 may be the basis of future therapeutic approaches to counteract cardiomyocyte hypertrophy. miRNAs are interesting targets for therapeutic use because they are selective. In fact they act on diseased tissues but they seem to have minimal effects on healthy tissues. Moreover, unlike their use in cardiovascular development, new delivery methods have been found, catheter-based delivery systems which allow the injury site to be directly targeted, bypassing effects on other tissue [150]. Figure 3 summarizes the main microRNAs involved in heart pathophysiology.

### 5.3. lncRNA in Heart Pathophysiology

miRNA are not the only ncRNAs implicated in regulatory processes. Recently another class of ncRNA, long non-coding RNA, so called because they are longer than 200 nt, has aroused much interest in cardiovascular function and disease (Table 2). lncRNAs are involved in cardiomyocyte differentiation, e.g., a new lncRNA (AK143260) was identified as a regulator of the cardiac lineage *in vitro*; it is required for mediating the transition from mesoderm to multipotent cardiac progenitors, regulating the activation of a network of cardiac differentiation specific genes [151]. Klattenhoff and colleagues named this lncRNA Braveheart because it is highly expressed in the heart, they identified Braveheart as a critical regulator of cardiovascular commitment from nascent mesoderm [15]. Recently, ANRIL, a multi-exonic lncRNA, has been shown to be implicated in epigenetic modulation in cardiac development and adult heart and also it has been associated with a locus implicated in cardiovascular disease [152,153]. Recently, it was shown that Fendrr, a lateral-mesoderm specific ncRNA, is fundamental in heart development in mouse. It mediates the epigenetic modification of target promoters thereby causing attenuation of the expression of transcription factors which are important in lateral mesoderm differentiation. Fendrr acts as chromatin modulator regulating PRC2 and TrxG/MLL, two histone-modifying complexes. PRC2 performs the methylation of histone H3 at lysine 27 whereas TrxG/MLL catalyzes the methylation at lysine 4; both are essential for embryonic development. Generally, the action of transcription factors is restricted in time and space; they have effects only in the cell in which they are expressed and for a limited time. Conversely, the epigenetic pattern can persist for different stages during differentiation. Fendrr follows this trend and it has a long-term effect [154]. Moreover the regulation operated by ncRNA might confer susceptibility to various diseases: e.g., a myocardial infarction-associated transcript (MIAT), also known as RNCR2 or Gomafu, is a long intergenic non-coding RNA that presents many genetic variants implicated in different processes. A large scale case-control association study regarding cardiovascular disease demonstrates that a MIAT variant (rs2301523) confers susceptibility to myocardial infarction [155]. However it is still unclear how this lincRNA acts. Another abundant class of ncRNA is Natural Antisense Transcript (NATs); they can derive from protein or non protein coding genes. NATs exhibit typical mRNAs properties, such as 3′ polyadenylation and 5′ cap but present very little sequence conservation [156]. Some NATs essential for heart function are implicated in regulation of the cardiac troponin I (cTNI) and myosin heavy chains (MHCs) and light chains. Various NATS of these genes have been identified in human and rat and all these have the ability to form sense-antisense RNA duplexes at different ratios during a life span, suggesting a role in the regulation of gene expression. However, the exact mechanism of repression used by NATs is to date unknown (reviewed in Luther 2005) [157]. Korostowski *et al*. found Kcnq1ot1, a new long ncRNA, on studying imprinting in the developing heart. Surprisingly the study revealed that Kcnq1 and Kcnq1ot1 lose their imprinted expression at the same time in the heart. However, Kcnq1ot1 regulates Kcnq1 transcription, not by regulating its imprinting, but through modulating chromatin flexibility and access to enhancers [16]. The increasing knowledge of endogenous antisense RNA will help us to understand better the mechanism of gene expression regulation. In summary, given the ever-expanding number of non-coding RNAs, understanding their function represents a formidable task. They can specifically target different genes, often in a one-to-many manner. Fine tuning the level of single ncRNA may therefore affect many pathways in a pleiotropic manner. New therapeutic strategies face the major challenge of developing standardized methods that combine high transfection efficiency with targeted delivery of miRNA to act on specific pathways. The new technologies might provide the ability to translate laboratory potential into clinical practice to prevent or treat cardiovascular diseases.

## 6. Conclusions

Non coding-RNAs critically affect the main molecular mechanisms involved in cardiovascular development and disease. It is well-established that dysregulation of miRNAs leads to development of vascular as well as cardiac disorders. Moreover, the signaling pathways regulated by miRNAs and miRNAs themselves could be potential therapeutic targets in cardiovascular diseases. Nevertheless, further investigation is necessary to define how the cross-talk between microRNAs and their targets can affect different physiological and pathological pathways in the cardiovascular system. Recently, increasing interest has been aroused by lncRNAs in cardiovascular research. Despite rapid progress in our understanding of lncRNAs, data on their role in cardiovascular patophysiology is still poor. Given that long ncRNAs are associated with several cellular processes, an improved understanding of the functional roles of long non-coding RNA is needed to identify new therapeutic targets in cardiovascular diseases. For this purpose, it will be interesting to investigate the expression of lncRNA in cardiovascular tissues and subsequently to define the mechanisms in which these molecules are involved.

## Figures and Tables

**Table 1:** Classes of non-coding RNAs (ncRNAs).

Non-coding RNAs	Symbol	Functions
Structural ncRNAs		

Transfer RNA	tRNA	mRNA translation
Ribosomal RNA	rRNA	mRNA translation

Regulatory ncRNA		
Short ncRNA		

Micro RNAs	miRNA	post-transcriptional regulators
PIWI-interacting RNA	piRNA	DNA methylation, transposon repression
Short interfering RNA	siRNA	RNA interference

Medium ncRNA		

Small nucleolar RNAs	snoRNA	RNA modification, rRNA processing
Promoter upstream transcripts	PROMPTs	Associated with chromatin changes
Transcription initiation RNAs	tiRNAs	Epigenetic regulation

Long ncRNAs		

Long intergenic ncRNA	lincRNAs	Epigenetic regulators of transcription
Enhancer-like ncRNA	eRNA	Transcriptional gene activation
Transcribed ultraconserved regions	T-UCRs	Regulation of miRNA and mRNA levels
Natural antisense transcripts	NATs	mRNA stability
Promoter-associated long RNAs	PALRs	chromatin changes
Pseudogenes	None	microRNA decoys

**Table 2:** Long-ncRNA in Heart Development and Pathophysiology.

Long-ncRNA	Function	Reference
**BraveHeart (AK143260)**	Cardiomyocytes differentiation	[15,151]
**ANRIL**	Epigenetic modulation in cardiac development.	[152,153]
**Fendrr**	Heart development	[154]
**MIAT**	Susceptibility to myocardial infarction.	[155]
**NATs**	Regulation of gene expression	[157]
**Kcnq1ot1**	Regulation of gene expression	[16]
